# The trend of suicide and self-harm in the Chinese population from 2018 to 2022 based on ambulance medical emergency cases: a retrospective study

**DOI:** 10.3389/fpubh.2025.1494841

**Published:** 2025-01-22

**Authors:** Ruizhe Yang, Jinsu Zhou, Francis Manyori Bigambo, Wu Yan, Xu Wang, Haibo Yang

**Affiliations:** ^1^Department of Public Health, Children's Hospital of Nanjing Medical University, Nanjing, China; ^2^Pediatric Intensive Care Unit, Department of Emergency, Children's Hospital of Nanjing Medical University, Nanjing, China; ^3^Pediatric Clinical Medical Research Center, Children's Hospital of Nanjing Medical University, Nanjing, China

**Keywords:** suicide, self-harm, ambulance medical emergency, epidemiology, Chinese population

## Abstract

**Objective:**

This study investigates the trends of suicide and self-harm in Nanjing, China, through 4 years of data collection, aiming to provide valuable information for developing effective suicide prevention strategies.

**Methods:**

This descriptive study analyzed Nanjing Emergency Medical Center (NEMC) ambulance records from Nanjing (2018–2022) to investigate suicide and self-harm events. Out of 689,305 records, 4,261 cases were included after exclusions. The study categorized incidents into 4,103 suicide events and 158 self-harm cases. Descriptive statistics and content analysis were conducted to identify characteristics and themes related to these events, with age groups defined according to American Medical Association standards.

**Results:**

The study highlights drug poisoning as the leading method, accounting for 63.56% of the 4,103 suicide events. It notes significant trends by age, gender, and season, with males showing higher rates of self-harm. The study emphasizes the need for targeted prevention strategies, particularly focusing on drug-related suicides among adults and adolescents, as well as the prevalence of various self-harming behaviors.

**Conclusion:**

To reduce self-harm and suicide, interventions must be strengthened for women, who experience higher rates. Key strategies include regulating pesticides and psychotropic drugs, increasing access to mental health resources, and launching community awareness campaigns. Additionally, training healthcare providers and promoting family education can enhance support for women facing mental health challenges.

## 1 Introduction

Suicide, defined as the intentional ending of one's own life (1), is a significant global public health issue that affects individuals across the lifespan and in diverse populations (2). According to recent estimates from the World Health Organization (WHO), more than 720,000 people die due to suicide every year. This makes suicide a significant global health issue and the third leading cause of death among individuals aged 15–29 years (3). The global average crude death rate is 9.2 per 100,000 population. Europe was the highest with a rate of 12.8 per 100,000 population, followed by Southeast Asia and the Americas (4). In addition, rates among men aged 45–64 years have increased from 21 suicides per 100,000 in 1999 to 30 per 100,000 in 2017 (5). Suicide has posed a serious challenge to the world, especially in low- and middle-income countries (6), where nearly 77% of suicide deaths occur (3).

Self-harm is often used to describe a broad spectrum of behaviors and intentions, including attempted hanging, impulsive self-poisoning, and superficial cutting, typically as a response to intolerable tension (7). Self-harm serves as a significant predictor of suicide (8–10). The risk is highest in the first 6 months following a self-harming episode but persists for several decades (10). Male gender, advanced age, and multiple instances of self-harm have been identified as predictors of subsequent suicide (9). Similar to suicide, self-harm rates fluctuate significantly among countries. In 2004, 5–9% of adolescents in Western countries reported having self-harmed (7). This illustrates the importance of analyzing the epidemiology of suicide and self-harm, as well as the related characteristics (11) to achieve the sustainable development goal of a one-third reduction in the global suicide rate by 2030 (12).

In China, alongside rapid economic development and changes in social structures, the rates of suicide and self-harm have significantly decreased among men and women, urban and rural residents, and across all age groups (13–15) over the past 20 years (15, 16). The WHO report estimated that the suicide rate in China decreased by 59.6%, from a standardized age-adjusted rate of 19.4 per 100,000 population in 2000 to 7.8 per 100,000 in 2012 (13). Globally, the overall incidence, mortality, and Disability-Adjusted Life Year (DALY) rates for self-harm showed a decreasing trend from 1999 to 2019, with estimated annual percentage changes (EAPC) of −1.5351, −2.0205, and −2.0605, respectively. This trend may also serve as evidence of a decline in self-harm rates in China (17). Explanations for this variation include differences in how suicide cases are classified, cultural attitudes toward suicide, access to lethal means, and the adequacy of treatment for mental disorders (5). Notably, although various Chinese provinces generally share a similar cultural background, the levels of their economic development and urbanization—factors closely related to suicide rates—vary significantly (18). Therefore, in order to precisely understand the trends of suicide and self-harm in China, studies that focus on representative regions and utilize high-quality medical registration data are indispensable (3).

Suicide and self-harm behaviors are multifactorial, involving complex interactions between biological and environmental determinants (19). In high-income countries, mental illnesses are estimated to be present in half of those who have died by suicide, with affective disorders (depression and bipolar disorder) accounting for one-third to half of all suicides (20). Studies of self-harmers presenting to hospitals using standard diagnostic criteria have shown that over 90% have at least one psychiatric disorder, most commonly depression, followed by substance abuse and anxiety disorders (21, 22). In China, ~30% of individuals who die by suicide have mental disorders, such as depression (23). Given the high prevalence and serious consequences of self-harm, it is also critical to further understand its risk and protective factors (24). Previous study has found that suicide rates are higher among older rural women, with pesticide ingestion being the most common method of suicide in China (25). Since the 1990s, restrictions on access to highly hazardous pesticides have contributed to a reduction in suicide deaths in China (26). In fact, the focus should shift to consider the driving forces behind suicide, particularly common risk factors such as socioeconomic disadvantage, low social support, increased burden, and unemployment, which have been increasingly associated with suicidal behaviors in recent years (27–29). Additionally, the epidemic of communicable diseases, such as COVID-19, can serve as a significant life stressor for individuals and those around them, profoundly impacting mental health, which should also be considered (30).

Nanjing, situated in Jiangsu Province within the Yangtze River Delta region, is not only characterized by its advanced industrial economy but also serves as a representative city of China (31). Nanjing has historically been a significant hub for culture, education, research, politics, economy, transportation networks, and tourism as well as the city exemplifies the demographic, cultural, and economic diversity characteristic of eastern China. In this study, we targeted this representative city in China to explore the reasons behind the shift in suicide and self-harm rates and trends. We aimed to investigate cases reported by the Nanjing Emergency Medical Center (NEMC) based on ambulance records from 2018 to 2022 and to provide strategies for future localized suicide and self-harm prevention.

## 2 Materials and methods

### 2.1 Data source

This descriptive study analyzed NEMC data on suicide and self-harm events based on ambulance call records among the Nanjing population from 2018 to 2022. As a provincial-level emergency medical rescue base, NEMC has a unified command and dispatch system for ambulances and a rapid response mechanism that has established a comprehensive rescue network. The need for consent to participate was deemed unnecessary and waived by the Institutional Ethics Committee (IEC) of the Children's Hospital of Nanjing Medical University (Approval No. 202309012-1) because the study utilized medical records and biological samples obtained from previous clinical treatments.

### 2.2 Participants

**Figure 1:**
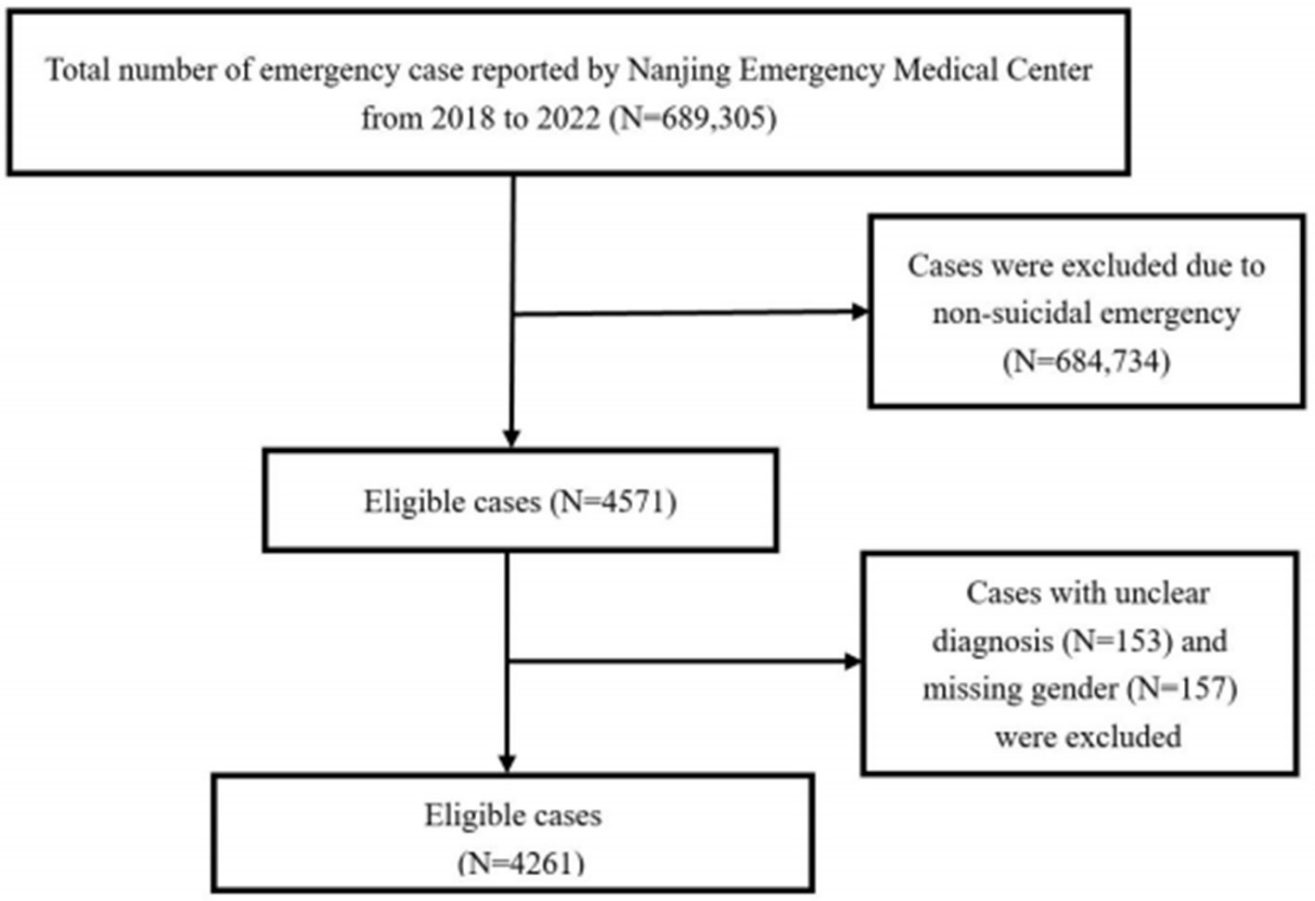
Flow diagram of suicide and self-harm cases screening, 2018–2022.

According to self-reported symptoms during ambulance calls, 689,305 records from the NEMC were initially reviewed by two authors (R.Y. and W.Y.). A total of 4,571 records related to self-harm and suicide events were identified during the study period. Of these incidents, 153 with missing gender information and 157 with unclear records were excluded during a double-check review by two additional authors (J.Z. and H.Y.), leaving 4,261 records included in the study. After reviewing these 4,261 records, two other authors (J.Z. and X.W.) classified 4,103 as suicide events and 158 as self-harm events (Figure 1).

### 2.3 Variables and analysis

Descriptive statistics were conducted for sex, age, report date, and method of self-harm or suicide. Three authors (R.Y., H.Y., and X.W.) performed a content analysis of self-reported symptoms and primary diagnoses to inductively identify characteristics and precipitating circumstances of self-harm and suicide, grouping them into themes for subgroup analysis. Following the American Medical Association's age designations, the age groups in this study include four sub-groups: Children (1–12 years), Adolescents (13–17 years), Adults (18 years and older), and Older Adults (65 years and older). A constant comparative approach was used to refine themes into more succinct categories (32). Discrepancies in coding were resolved and data saturation was confirmed by reaching a mutual consensus after a discussion between authors (32). Statistical analyses were performed with SPSS, version 26.0 (IBM Corp).

## 3 Result

Table 1 present a sobering overview of suicide methods based on 4,103 reported events during 2018–2022, revealing concerning trends, particularly in drug poisoning, which accounts for 2,608 events (63.56%). Cutting wrists is the second most common method, with 626 events (15.26%), emphasizing the importance of recognizing self-harming behaviors that can lead to fatal outcomes, while hanging, reported in 509 events (12.41%), remains a notable method despite being less frequent than drug poisoning and wrist cutting. Falling, with 128 events (3.12%), is less common than the top three methods but still significant, whereas drowning, noted in only 41 events (1.00%), and gas poisoning, with 38 events (0.93%), are among the least common methods, indicating they may not be primary focuses for suicide prevention efforts. Additionally, various unspecified methods total 69 events (1.68%), and the use of multiple methods in 84 events (2.05%).

**Table 1:** Suicide cases reported by Nanjing Emergency Medical Center in Nanjing, Jiangsu, 2018–2022.

**Suicide methods**	**Cases *N* (%)^a^**
Hanging	509 (12.41%)
Drowning	41 (1.00%)
Falling	128 (3.12%)
Cutting wrists	626 (15.26%)
Drug poisoning	2,608 (63.56%)
Gas poisoning	38 (0.93%)
Other methods	69 (1.68%)
Multiple methods	84 (2.05%)
Total	4,103

^a^Percentage is counted out of total observed cases.

During the reporting period, a total of 158 self-harm events were recorded, categorized into four primary methods. Body injury accounted for the highest number of incidents, with 103 events (65.19%), including behaviors like hitting oneself and burning, indicating its prevalence among individuals seeking emergency medical assistance in Nanjing. Other unspecified self-harming behaviors totaled 45 events (28.48%), reflecting a diversity of practices. Cutting wrists was reported in 7 events (4.43%), making it less common, while swallowing foreign bodies was noted in 3 events (1.90%). Overall, the data highlights body injury as the most prevalent form of self-harm, with a significant variety of methods suggesting a need for further exploration and tailored support for those engaging in these behaviors (Table 2).

**Table 2:** Self-harm cases reported by Nanjing Emergency Medical Center in Nanjing, Jiangsu, 2018–2022.

**Self-harm methods**	**Cases *N* (%)^a^**
Cutting wrists	7 (4.43%)
Body injury	103 (65.19%)
Swallow foreign bodies	3 (1.90%)
Other methods	45 (28.48%)
Total	158

^a^Percentage is counted out of total observed cases.

**Figure 2:**
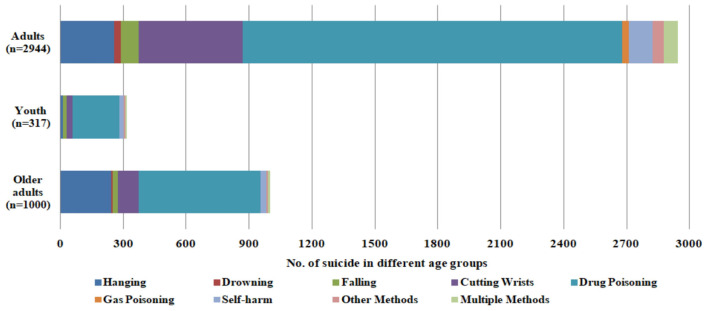
Age group associate with suicide methods of case reported by Nanjing Emergency Medical Center, 2018–2022.

Based on confirmed suicide and self-harm methods in the study, four subgroups were created by different themes, including (1) Years, (2) Seasons, (3) Age, and (4) Gender. For suicide (Table 3 and Figure 2), the age group indicated adult population remained the most reported events compared with youth and older adult populations, and drug poisoning had the largest proportion. In fact, drug poisoning dominated in all three age groups, while hanging shared a similar proportion among the adult and older adult populations rather than that in the youth population. Boundaries of various themes are usually affected due to the complexity associated with interactions between environmental factors and individual characteristics, such as drug poisoning caused most suicide events in all four seasons as same as the trend from 2018 to 2022.

**Table 3:** Individual characteristics of suicide cases reported by Nanjing Emergency Medical Center in Nanjing, Jiangsu, 2018–2022.

**Characteristics**	**Cases *N* (%)^a^**
**Year**	
2018	374 (9.12%)
2019	831 (20.25%)
2020	1,021 (24.88%)
2021	1,101 (26.83%)
2022	776 (18.91%)
**Sex**	
Male	1,547 (37.70%)
Female	2,556 (62.30%)
**Season** ^b^	
Spring	1,102 (26.86%)
Summer	1,182 (28.81%)
Fall	968 (23.59%)
Winter	851 (20.74%)
**Age group** ^c^	
Children	31 (0.76%)
Adolescents	268 (6.53%)
Adults	2,831 (69.00%)
Older adults	973 (23.71%)

^a^Percentage is counted out of total observed cases.

^b^Spring runs from March 1 to May 31; Summer runs from June 1 to August 31; Fall runs from September 1 to November 30; and Winter runs from December 1 to February 28 (February 29 in a leap year).

^c^The following are the American Medical Associations' age designations: Children (1–12 years); Adolescents (13–17 years); Adults (18 years or older); and Older adults (65 and older).

**Figure 3:**
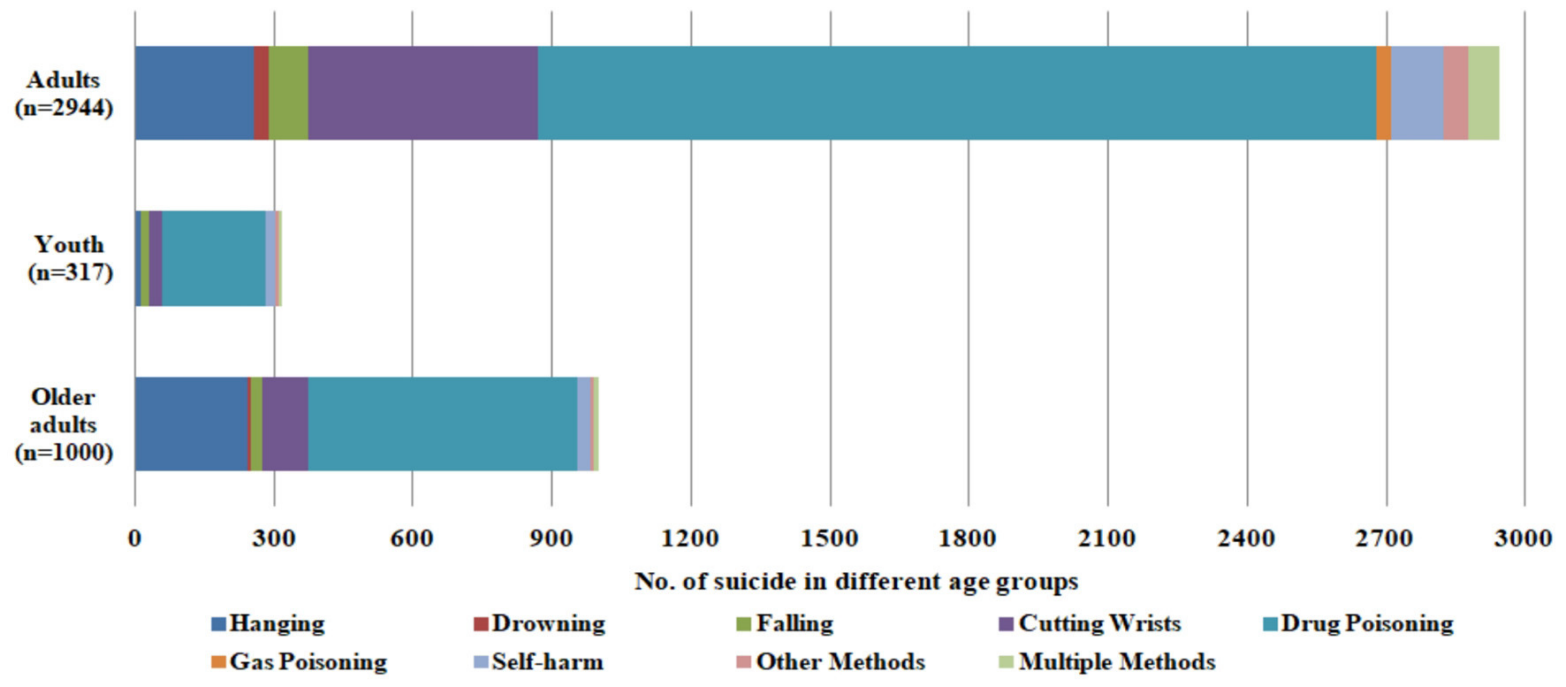
Age group associate with suicide methods of case reported by Nanjing Emergency Medical Center, 2018–2022. The following are the American Medical Associations' age dessignations: youth (1–17 year old); Adults (18 years or olders) and older adults (65 and older).

The trends observed in Table 4 and Figure 3 of self-harm incidents in Nanjing from 2018 to 2022 discloses prominent trends. There is a distinct preponderance of self-harm among males over females, suggesting that societal attitudes might influence emotional expression. Seasonal variations indicate higher occurrences in spring and fall, while lower rates are observed in summer and winter, suggesting that environmental factors could play a role. Most self-harm incidents occur among adults, with few among adolescents and none reported among children. These findings emphasize the complexity of self-harm behaviors and the necessity for targeted interventions considering gender, seasonal influences, and age-related factors.

**Table 4:** Individual characteristics of self-harm cases reported by Nanjing Emergency Medical Center in Nanjing, Jiangsu, 2018–2022.

**Characteristics**	**Cases *N* (%)^a^**
**Year**	
2018	14 (8.86%)
2019	34 (21.52%)
2020	51 (32.28%)
2021	37 (23.42%)
2022	22 (13.92%)
**Sex**	
Male	92 (58.23%)
Female	66 (41.77%)
**Season** ^b^	
Spring	49 (31.01%)
Summer	31 (19.62%)
Fall	42 (26.58%)
Winter	36 (22.78%)
**Age group** ^c^	
Children	0 (0.00%)
Adolescents	18 (11.39%)
Adults	113 (71.52%)
Older adults	27 (17.09%)

^a^Percentage is counted out of total observed cases.

^b^Spring runs from March 1 to May 31; Summer runs from June 1 to August 31; Fall runs from September 1 to November 30; and Winter runs from December 1 to February 28 (February 29 in a leap year).

^c^The following are the American Medical Associations' age designations: Children (1–12 years); Adolescents (13–17 years); Adults (18 years or older); and Older adults (65 and older).

### 3.1 Years

Table 5 shows 3,103 reported suicide events, increasing from 374 in 2018 to a peak of 1,101 in 2021, then declining to 776 in 2022. Drug poisoning was the most common method, accounting for 62.03% of events in 2018 and peaking at 66.76% in 2021, before slightly decreasing to 64.56% in 2022. This method consistently represented the majority over the 5 years.

**Table 5:** Suicide cases reported by Nanjing Emergency Medical Center from 2018 to 2022 in Nanjing, Jiangsu.

		**Year**				
		**2018**	**2019**	**2020**	**2021**	**2022**
Suicide methods, *N* (%)^a^	Hanging	47 (12.57%)	107 (12.88%)	140 (13.71%)	120 (10.90%)	95 (12.24%)
	Drowning	4 (1.07%)	12 (1.44%)	11 (1.08%)	8 (0.73%)	6 (0.77%)
	Falling	11 (2.94%)	25 (3.01%)	38 (3.72%)	30 (2.72%)	24 (3.09%)
	Cutting wrists	52 (13.90%)	152 (18.29%)	152 (14.89%)	163 (14.80%)	107 (13.79%)
	Drug poisoning	232 (62.03%)	505 (60.77%)	635 (62.19%)	735 (66.76%)	501 (64.56%)
	Gas poisoning	5 (1.34%)	7 (0.84%)	8 (0.78%)	11 (1.00%)	7 (0.90%)
	Other methods	12 (3.21%)	12 (1.44%)	14 (1.37%)	14 (1.27%)	17 (2.19%)
	Multiple methods	11 (2.94%)	11 (1.32%)	23 (2.25%)	20 (1.82%)	19 (2.45%)
	Total	374	831	1,021	1,101	776

^a^Percentage is counted out of total observed cases per year.

Hanging was the second most common method, starting at 12.57% in 2018, peaking at 13.71% in 2020, dropping to 10.90% in 2021, and rising to 12.24% in 2022. Cutting wrists was significant in 2019 and 2020, reaching 18.29% and 14.89%, respectively, but declined to 13.79% by 2022. Other methods, like drowning, falling, gas poisoning, and multiple methods, accounted for smaller percentages, with drowning remaining below 2% each year.

### 3.2 Seasons

The highest number occurring in the summer (1,182 events) and the lowest in winter (851 events) (Table 6). The predominant method of suicide across all seasons is drug poisoning, accounting for ~63%−64% of events, with a notable peak in summer (759 events, 64.21%). Cutting wrists is the second most common method, consistently representing around 15% of events in each season, with a slight decrease in winter (134 events, 15.75%). Hanging is also a significant method, particularly in spring (146 events, 13.25%) and summer (152 events, 12.86%), while drowning remains the least common method, with the highest incidence in spring (15 events, 1.36%). Other methods, including falling, gas poisoning, and multiple methods, show varying frequencies, with falling being more prevalent in the fall (39 events, 4.03%).

**Table 6:** Suicide cases in different seasons^b^ reported by Nanjing Emergency Medical Center, Nanjing, Jiangsu, 2018–2022.

		**Season**			
		**Spring**	**Summer**	**Fall**	**Winter**
Suicide methods, *N* (%)^a^	Hanging	146 (13.25%)	152 (12.86%)	107 (11.05%)	104 (12.22%)
	Drowning	15 (1.36%)	11 (0.93%)	6 (0.62%)	9 (1.06%)
	Falling	26 (2.36%)	39 (3.30%)	39 (4.03%)	24 (2.82%)
	Cutting wrists	175 (15.88%)	171 (14.47%)	146 (15.08%)	134 (15.75%)
	Drug poisoning	694 (62.98%)	759 (64.21%)	620 (64.05%)	535 (62.87%)
	Gas poisoning	12 (1.09%)	9 (0.76%)	9 (0.93%)	8 (0.94%)
	Other methods	17 (1.54%)	19 (1.61%)	17 (1.76%)	16 (1.88%)
	Multiple methods	17 (1.54%)	22 (1.86%)	24 (2.48%)	21 (2.47%)
	Total	1,102	1,182	968	851

^a^Percentage is counted out of total observed cases in different seasons.

^b^Spring runs from March 1 to May 31; Summer runs from June 1 to August 31; Fall runs from September 1 to November 30; and Winter runs from December 1 to February 28 (February 29 in a leap year).

### 3.3 Age groups

Table 7 presents an analysis of suicide events by age group, as reported by the Nanjing Emergency Medical Center in Jiangsu, China, from 2018 to 2022. The data is divided into four age groups: children, adolescents, adults, and older adults. In total, there were 31 events for children, 268 for adolescents, 2,831 for adults, and 973 for older adults.

**Table 7:** Suicide cases of different age groups^b^ reported by Nanjing Emergency Medical Center in Nanjing, Jiangsu, 2018–2022.

		**Age group**			
		**Children**	**Adolescents**	**Adults**	**Older adults**
Suicide methods, *N* (%)^a^	Hanging	4 (12.90%)	8 (2.99%)	256 (9.04%)	241 (24.77%)
	Drowning	0 (0.00%)	2 (0.75%)	32 (1.13%)	7 (0.72%)
	Falling	2 (6.45%)	13 (4.85%)	85 (3.00%)	28 (2.88%)
	Cutting wrists	0 (0.00%)	31 (11.57%)	496 (17.52%)	99 (10.17%)
	Drug poisoning	24 (77.42%)	196 (73.13%)	1,808 (63.86%)	(580 59.61%)
	Gas poisoning	0 (0.00%)	3 (1.12%)	35 (1.24%)	0 (0.00%)
	Other methods	1 (3.23%)	7 (2.61%)	52 (1.84%)	9 (0.92%)
	Multiple methods	0 (0.00%)	8 (2.99%)	67 (2.37%)	9 (0.92%)
	Total	31	268	2,831	973

^a^Percentage is counted out of total observed cases in each age group.

^b^The following are the American Medical Associations' age designations: Children (1–12 years); Adolescents (13–17 years); Adults (18 years or older); and Older adults (65 and older).

The most common method of suicide across all age groups was drug poisoning, which accounted for a significant percentage of events: 77.42% for children, 73.13% for adolescents, 63.86% for adults, and 59.61% for older adults. This indicates a concerning trend of high drug-related suicides, particularly among younger individuals.

Hanging was the second most common method, especially among adults (9.04%) and older adults (24.77%), but it was less common among children (12.90%) and adolescents (2.99%). Drowning and falling were reported less frequently, with drowning being particularly rare among children (0.00%) and older adults (0.72%). Cutting wrists was notable among adolescents (11.57%) and adults (17.52%), but not among children. The categories of “other methods” and “multiple methods” accounted for a small portion of events in all age groups, with the highest being 3.23% for “other methods” among children and 2.99% for “multiple methods” among adolescents.

### 3.4 Gender

A total of 4,103 suicide events were reported, with 1,547 involving males and 2,556 involving females. The most common method of suicide among males was drug poisoning, accounting for 56.17% (869 events), followed by hanging at 21.01% (325 events) and wrist cutting at 11.12% (172 events). In contrast, females showed a higher prevalence of wrist cutting, which constituted 17.76% (454 events) of their total, while drug poisoning was also significant at 68.04% (1,739 events).

Other methods included hanging, drowning, and gas poisoning. Males had a higher percentage of hanging incidents (21.01%) compared to females (7.20%). Drowning was relatively uncommon, with males at 1.36% (21 events) and females at 0.78% (20 events). Falling incidents were slightly more frequent among males (4.20%) than females (2.46%). Gas poisoning was infrequent, with males at 1.55% (24 events) and females at 0.55% (14 events) (Table 8).

**Table 8:** Suicide cases of sexual group reported by Nanjing Emergency Medical Center in Nanjing, Jiangsu, 2018–2022.

		**Sexual group**	
		**Male**	**Female**
Suicide methods, *N* (%)^a^	Hanging	325 (21.01%)	184 (7.20%)
	Drowning	21 (1.36%)	20 (0.78%)
	Falling	65 (4.20%)	63 (2.46%)
	Cutting wrists	172 (11.12%)	454 (17.76%)
	Drug poisoning	869 (56.17%)	1,739 (68.04%)
	Gas poisoning	24 (1.55%)	14 (0.55%)
	Other methods	41 (2.65%)	28 (1.10%)
	Multiple methods	30 (1.94%)	54 (2.11%)
	Total	1,547	2,556

^a^Percentage is counted out of total observed cases in each sexual group.

### 3.5 Classification of drug poisoning

In this study, we focused significantly on drug poisoning as the leading method of suicide among the sample population, classifying drug types based on the chief complaints of patients or descriptions provided by those who called for emergency assistance (Table 9). A total of 1,197 cases (44.97%) involved overdoses of psychotropic medications, including sleeping pills, antidepressants, and the sedative lorazepam. Additionally, overdoses of other drugs, such as cold medications, hypotensive drugs, and specific medications for other conditions, accounted for 31.56% of cases. For example, one case involved an 85-year-old female who “took valsartan 80 mg, ~40 capsules over more than 2 h,” while another case involved a 29-year-old male who “drank toilet cleaner for 40 min.” Notably, suicides involving pesticides accounted for 21.30%, placing them in third position, followed by rat poison (2.07%) and opioids (0.11%).

**Table 9:** Classification of drug poisoning in reported suicide cases.

**Classification of drug poisoning**	**Cases *N* (%)^a^**
Pesticide	567 (21.30)
Rat poison	55 (2.07)
Opioids	3 (0.11)
Psychotropic medicine	1,197 (44.97)
Other drugs	840 (31.56)
Total	2,662

^a^Percentage is counted out of total drug poisoning cases.

## 4 Discussion

This descriptive study examined ambulance records from the NEMC between 2018 and 2022, focusing on suicide and self-harm events. Out of 689,305 calls, 4,261 cases related to self-harm or suicide were analyzed. The study found that drug poisoning was the most prevalent method of suicide, accounting for 63.56% of cases, followed by wrist cutting (15.26%) and hanging (12.41%). Self-harm incidents primarily involved body injuries (65.19%). The analysis revealed significant trends across age groups, seasons, and gender, with higher rates among adults and males. Drug poisoning was notably common across all age groups, particularly among younger individuals.

Our study revealed that both men and adults were the two main groups that endured higher burdens of self-harm compared to others. Although the national circumstances in China indicated that females used to be in disadvantaged positions and had greater burdens of self-harm than males (33). Nevertheless, our findings indicated that females witnessed a greater reduction in the burden of self-harm than males from 2018 to 2022 in Nanjing, which was consistent with the national results in China (14, 34, 35). Some researches attributed the big declining trends among females to the process of urbanization and economic development during the past decades (36). Meanwhile, in the current social changes in China, intense work stress and anxiety caused by the rapid increase in the cost of living may exert more pressure on males. These factors are not beneficial to their mental health and make them prone to mental illness, a key risk factor for self-harm (37).

According to previous research, although suicide has been a challenging public health problem in China, (38) with the rising economic levels and great social changes, a dramatic decline in Chinese suicide rates has been observed in the last three decades (16, 39, 40). Our study reported that the previously declining trend in suicides has recently reversed in Nanjing. To the best of our knowledge, these findings are consistent with a recent study of a large nationwide sample in China (2), which indicated a relatively high prevalence of suicidal idealization among the general population during the COVID-19 pandemic. The outbreak of the pandemic has had profound health, psychological, social, and economic consequences worldwide, which may have heightened various suicide risk factors (41, 42). Nevertheless, the number of suicide cases observed in 2022 has reduced to a similar level in 2019. This indicates that as the pandemic evolves, the suicide situation might be changing, so it is necessary to have ongoing monitoring and real-time surveillance (43).

Our study revealed that the seasonal peak of suicidal behaviors was typically witnessed in summer and hit the bottom in winter. A previous study examined the influence of social and bio-climatic factors by using 28 countries time series data, reported that populations in the temperature zone exhibited suicide seasonality (44). The climate in the temperate zone always showed the widest seasonal change with the longest day length in spring time/early summer which may introduce a potential influence from a geographic perspective toward the seasonal cycle of suicides (45). The activity of serotonin that regulated the mood and impulse control of human beings were sensitive to climate change and a marked seasonal fluctuation was reported (46). It was noted that the malfunctioning of one's serotonin could be one of the most imperative factors that drive a person to have a deliberate self-harm behavior (47, 48). The inter-correlation among climate change, serotonin function, and suicidal ideation may help explain the seasonal effect on suicides, as studies show a strong link between climatic factors, especially sunshine exposure, and suicidal behavior (45, 49, 50).

However, this might not hold true for those countries situated in the tropical zone where the climate exhibits no distinct seasons. No seasonal pattern was discerned in suicide deaths in Colombia, a country located in the inter-tropical zone with stable temperatures throughout the year (51). A similar finding was reported in the city of Sao Paulo which is a municipality in the southeast region of Brazil (52) and in Singapore, one of the Asian countries lying one degree north of the equator (53). A study by Cantor et al. (54) examined the Caucasian population near the equator in Australia. It found that male suicides peaked in spring and early summer, while female suicides were at their lowest in autumn. Although some variations were noted, climate factors appeared to have less influence in the tropical zone.

These findings highlight a clear seasonal pattern in suicidal behaviors, which should impact existing public health measures mentioned previously. For instance, it may be beneficial to reinforce connections with patients who have a history of suicide attempts or suicidal thoughts during the summer through brief contact interventions (55, 56). In addition, broad public health campaigns for prevention and awareness should be implemented during at-risk periods. Practitioners should also be more vigilant in the spring and summer for patients at risk of suicide, including those with suicidal ideation, a history of suicidal behaviors, or psychiatric disorders (57). Nevertheless, further studies are needed to better understand the physiological mechanisms underlying the meteorological factors that impact suicidal behaviors (57).

The increase in suicide cases seen in this study is mainly due to rising rates among young and older adults. In China, suicide is the leading cause of death for people aged 15–34, significantly adding to the overall health burden (58). Moreover, stress-diathesis models suggest that stressful life events interact with vulnerability factors, increasing the likelihood of suicidal behavior (59). Adolescence can be a vulnerable time for dealing with life stressors like family conflicts and academic pressure (60). Previous studies found that many university students do not value life enough and have a weak sense of survival, which may indicate a lack of social support and hope among young people (61). In fact, studies found that depressive symptoms had direct and indirect effects on suicide risk among young adults (62, 63), however, these correlations were weak (61). Thus, many young people at risk of suicide may not have mood disorder while the risk come from not valuing life, having poor problem-solving skills, and other reasons, like believing that suicide is a way to express or defend their faith (61). Regarding young adults in Nanjing, concern about economic problems is a common trigger for suicide among this age group (64), Many young adults play the role of breadwinners for their families and consequently experience more economic stress than people in other age groups (18).

On the other hand, China is experiencing a rapidly aging population as the first wave of baby boomers born in the 1950s and 1960s began turning 60 in the 2010s (65). Rapid macro-socio-economic changes and urbanization may lead to disconnection, physical alienation, loss of normal social interactions, and increased anxiety or depression among the older population in Nanjing (66, 67). Moreover, suicidal behavior is associated with chronic diseases which were significant in older population (68). The modernization of a society leads to dramatic social changes that without doubt would affect older people (46). A higher life expectancy leads to a larger proportion of older adult people in the population, raising concerns about resource competition. Research suggests that a well-developed social welfare system for the older adult is a key protective factor against suicide among older adults. Enhancing social activities for this group may foster connections and reduce the seasonal effects related to their means of staying in touch. A better social welfare system, along with proactive engagement with older adults, could significantly improve the current situation (46).

Regarding gender, previous studies have indicated that females have higher suicidal ideation than males (69, 70), which is consistent with our findings as well. Although one of studies from South Korea have not reached a definite conclusion (71), additional has proved that female with low socio-economic status have a significantly higher number of suicide attempts, especially those who are receiving national basic livelihood security (OR: 1.820) (72). However, more studies have found that female suicide is related to increased economic stress, unemployment, and income loss (73, 74). Specifically, lifetime incarceration, previous trauma, and earning < $40,000 emerged as a greater risk for suicide attempts in women (75). Data reported by the WHO in 2012 align with our study results, indicating that female students in China had higher suicide rates (61). Therefore, it is important to further explore how gender differences affect suicide risk.

Lastly, ingesting pesticides was once the most common method of suicide in China, accounting for 58% of all suicides between 1996 and 2000 (76). However, after strict laws were implemented on the use of lethal pesticides in agriculture, the rate of pesticide-related suicides declined rapidly (77). Today, drug poisoning, especially the misuse of mental health and sedative drugs, has emerged as the most common means of suicide in Nanjing. This suggests that suicide prevention strategies should be updated (3). In one respect, the increase in depression diagnoses among suicide victims indicates that more attention should be paid to the treatment of depression (3). For instance, China's primary mental health care system is hospital-centered and needs improvement to better cover high-risk populations (78), and the government should enhance the regulation of psychotropic drugs through both policy and legal means.

A key advantage of this study lies in the fact that we employed suicide data from the NEMC, which covers all emergency calls in Nanjing. Such data is highly representative and avoids the risk of sampling errors. However, this study also has some limitations. First, it was a cross-sectional study, and the causal relationship between multiple risk factors could not be determined (2). Second, suicide is a sensitive subject in China, which implies that additional demographic details, such as educational level, employment status, and medical complications, are frequently not accessible. Therefore, without understanding the main factors that lead to suicide attempts, we cannot effectively target prevention efforts (79). The suicide cases in our study did not include regional information about where the suicides occurred, which may underestimate the number of cases, particularly in rural areas, and makes analysis more difficult.

## 5 Conclusion

In summary, the number of self-harm and suicide cases among ambulance medical emergencies in Nanjing, China, rose from 2018 to 2021 but declined in 2022. Females, as well as young and older adults, were particularly impacted, with incidents happening most frequently in summer. Drug poisoning was the most prevalent method of suicide. These findings enhance our comprehension of self-harm and suicide trends, assisting in identifying at-risk populations and informing targeted prevention strategies. Additionally, future research should incorporate longitudinal studies that broaden the measurement of self-harm and suicide behaviors and systematically evaluate long-term outcomes.

## Data Availability

The raw data supporting the conclusions of this article will be made available after notifying corresponding author *via* email. Requests to access the datasets should be directed to Xu Wang, sepnine@njmu.edu.cn.

## References

[1] Rogers JP Chesney E Oliver D Begum N Saini A Wang S. Suicide, self-harm and thoughts of suicide or self-harm in infectious disease epidemics: a systematic review and meta-analysis. Epidemiol Psychiatr Sci. (2021) 30:e32. 10.1017/S2045796021000354 33902775 PMC7610720

[2] Shi L Que J-Y Lu Z-A Gong Y-M Liu L Wang Y-H. Prevalence and correlates of suicidal ideation among the general population in China during the COVID-19 pandemic. Eur Psychiatry. (2021) 64:e18. 10.1192/j.eurpsy.2021.5 33686933 PMC7943957

[3] Qiao J Xia T Fang B Cai R Chen L Qian N. The reversing trend in suicide rates in Shanghai, China, from 2002 to 2020. J Affect Disord. (2022) 308:147–154. 10.1016/j.jad.2022.04.056 35429532

[4] Stone DM Simon TR Fowler KA Kegler SR Yuan K Holland KM. Vital signs: trends in state suicide rates—United States, 1999-2016 and circumstances contributing to suicide-−27 States, 2015. MMWR Morb Mortal Wkly Rep. (2018) 67:617–624. 10.15585/mmwr.mm6722a1 29879094 PMC5991813

[5] Fazel S Runeson B. Suicide. N Engl J Med. (2020) 382:266–274. 10.1056/NEJMra1902944 31940700 PMC7116087

[6] Phillips MR Cheng HG. The changing global face of suicide. Lancet. (2012) 379:2318–9. 10.1016/S0140-6736(12)60913-1 22726503

[7] Skegg K. Self-harm. Lancet. (2005) 366:1471–83. 10.1016/S0140-6736(05)67600-3 16243093

[8] Suominen K Isometsä E Suokas J Haukka J Achte K Lönnqvist J. Completed suicide after a suicide attempt: a 37-year follow-up study. Am J Psychiatry. (2004) 161:562–3. 10.1176/appi.ajp.161.3.562 14992984

[9] Cooper J Kapur N Webb R Lawlor M Guthrie E Mackway-Jones K. Suicide after deliberate self-harm: a 4-year cohort study. Am J Psychiatry. (2005) 162:297–303. 10.1176/appi.ajp.162.2.297 15677594

[10] Zahl DL Hawton K. Repetition of deliberate self-harm and subsequent suicide risk: long-term follow-up study of 11,583 patients. Br J Psychiatry. (2004) 185:70–5. 10.1192/bjp.185.1.70 15231558

[11] Zou Y Leung R Lin S Yang M Lu T Li X. Attitudes towards suicide in urban and rural China: a population based, cross-sectional study. BMC Psychiatry. (2016) 16:162. 10.1186/s12888-016-0872-z 27230910 PMC4881201

[12] Yip PSF Zheng Y Wong C. Demographic and epidemiological decomposition analysis of global changes in suicide rates and numbers over the period 1990-2019. Inj Prev. (2022) 28:117-124. 10.1136/injuryprev-2021-044263 34400542

[13] Sha F Chang Q Law YW Hong Q Yip PSF. Suicide rates in China, 2004-2014: comparing data from two sample-based mortality surveillance systems. BMC Public Health. (2018) 18:239. 10.1186/s12889-018-5161-y 29433460 PMC5809896

[14] Wang CW Chan CL Yip PS. Suicide rates in China from 2002 to 2011: an update. Soc Psychiatry Psychiatr Epidemiol. (2014) 49:929–41. 10.1007/s00127-013-0789-5 24240568

[15] Zhang J Sun L Liu Y Zhang J. The change in suicide rates between 2002 and 2011 in China. Suicide Life Threat Behav. (2014) 44:560–8. 10.1111/sltb.12090 24690079

[16] Yip PSF Liu KY Hu J Song XM. Suicide rates in China during a decade of rapid social changes. Soc Psychiatry Psychiatr Epidemiol. (2005) 40:792–8. 10.1007/s00127-005-0952-8 16205852

[17] Zhou X Li R Cheng P Wang X Gao Q Zhu H. Global burden of self-harm and interpersonal violence and influencing factors study 1990–2019: analysis of the global burden of disease study. BMC Public Health. (2024) 24:1035. 10.1186/s12889-024-18151-3 38614987 PMC11016221

[18] Cai Z Chen M Ye P Yip PSF. Socio-economic determinants of suicide rates in transforming China: A spatial-temporal analysis from 1990 to 2015. Lancet Reg Health West Pac. (2022) 19:100341. 10.1016/j.lanwpc.2021.100341 35024666 PMC8671725

[19] van Heeringen K Mann JJ. The neurobiology of suicide. Lancet Psychiatry. (2014) 1:63–72. 10.1016/S2215-0366(14)70220-2 26360403

[20] Arsenault-Lapierre G Kim C Turecki G. Psychiatric diagnoses in 3275 suicides: a meta-analysis. BMC Psychiatry. (2004) 4:37. 10.1186/1471-244X-4-37 15527502 PMC534107

[21] Haw C Hawton K Houston K Townsend E. Psychiatric and personality disorders in deliberate self-harm patients. Br J Psychiatry. (2001) 178:48–54. 10.1192/bjp.178.1.48 11136210

[22] Suominen K Henriksson M Suokas J Isometsä E Ostamo A Lönnqvist J. Mental disorders and comorbidity in attempted suicide. Acta Psychiatr Scand. (1996) 94:234–40. 10.1111/j.1600-0447.1996.tb09855.x 8911558

[23] Zhang J Li Z. Suicide means used by Chinese rural youths: a comparison between those with and without mental disorders. J Nerv Ment Dis. (2011) 199:410–5. 10.1097/NMD.0b013e31821d3ac7 21629021 PMC3205915

[24] Ma Y Li Y Xie X Zhang Y Ammerman BA Lewis SP. The role of depressive symptoms and social support in the association of internet addiction with non-suicidal self-injury among adolescents: a cohort study in China. BMC Psychiatry. (2023) 23:322. 10.1186/s12888-023-04754-4 37161436 PMC10169141

[25] Zhang X Li H-S Zhu Q-H Zhou J Zhang S Zhang L. Trends in suicide by poisoning in China 2000-2006: age, gender, method, and geography. Biomed Environ Sci. (2008) 21:253–6. 10.1016/S0895-3988(08)60038-0 18714825

[26] Page A Liu S Gunnell D Astell-Burt T Feng X Wang L. Suicide by pesticide poisoning remains a priority for suicide prevention in China: Analysis of national mortality trends 2006–2013. J Affect Disord. (2017) 208:418–23. 10.1016/j.jad.2016.10.047 27842298

[27] Iob E Steptoe A Fancourt D. Abuse, self-harm and suicidal ideation in the UK during the COVID-19 pandemic. Br J Psychiatry. (2020) 217:543–6. 10.1192/bjp.2020.130 32654678 PMC7360935

[28] Gratz KL Tull MT Richmond JR Edmonds KA Scamaldo KM Rose JP. Thwarted belongingness and perceived burdensomeness explain the associations of COVID-19 social and economic consequences to suicide risk. Suicide Life Threat Behav. (2020) 50:1140–8. 10.1111/sltb.12654 32589811 PMC7361587

[29] Li DJ Ko NY Chen YL Wang PW Chang YP Yen CF. COVID-19-related factors associated with sleep disturbance and suicidal thoughts among the Taiwanese Public: a Facebook survey. Int J Environ Res Public Health. (2020) 17:4479. 10.3390/ijerph17124479 32580433 PMC7345275

[30] Tucci V Moukaddam N Meadows J Shah S Galwankar SC Kapur GB. The forgotten plague: psychiatric manifestations of Ebola, Zika, and emerging infectious diseases. J Glob Infect Dis. (2017) 9:151–6. 10.4103/jgid.jgid_66_17 29302150 PMC5750439

[31] Yang H Ge A Xie H Li W Qin Y Yang W. Effects of ambient air pollution on precocious puberty: a case-crossover analysis in Nanjing, China. J Clin Med. (2022) 12:282. 10.3390/jcm12010282 36615082 PMC9821251

[32] Ruch DA Heck KM Sheftall AH Fontanella CA Stevens J Zhu M. Characteristics and precipitating circumstances of suicide among children aged 5 to 11 years in the United States, 2013–2017. JAMA Netw Open. (2021) 4:e2115683. 10.1001/jamanetworkopen.2021.15683 34313741 PMC8317003

[33] Liu S Page A Yin P Astell-Burt T Feng X Liu Y. Spatiotemporal variation and social determinants of suicide in China, 2006–2012: findings from a nationally representative mortality surveillance system. Psychol Med. (2015) 45:3259–68. 10.1017/S0033291715001269 26138093

[34] Haagsma JA Graetz N Bolliger I Naghavi M Higashi H Mullany EC. The global burden of injury: incidence, mortality, disability-adjusted life years and time trends from the Global Burden of Disease study 2013. Inj Prev. (2016) 22:3–18. 10.1136/injuryprev-2015-041616 26635210 PMC4752630

[35] Wang Z Wang J Bao J Gao X Yu C Xiang H. Temporal trends of suicide mortality in mainland China: results from the age-period-cohort framework. Int J Environ Res Public Health. (2016) 13:784. 10.3390/ijerph13080784 27527195 PMC4997470

[36] Sha F Yip PS Law YW. Decomposing change in China's suicide rate, 1990–2010: ageing and urbanisation. Inj Prev. (2017) 23:40–5. 10.1136/injuryprev-2016-042006 27312962

[37] Pan J Zhang L Tang Y Li Q Yu C He T. Sharply reduced but still heavy self-harm burdens in Hubei Province, China, 1990–2015. Int J Environ Res Public Health. (2018) 15:391. 10.3390/ijerph15020391 29495306 PMC5858460

[38] Yu B. Suicide in China: the power of social and economic change. Lancet Reg Health West Pac. (2022) 19:100356. 10.1016/j.lanwpc.2021.100356 35024671 PMC8733181

[39] Chen X Sun Y Li Z Yu B Gao G Wang P. Historical trends in suicide risk for the residents of mainland China: APC modeling of the archived national suicide mortality rates during 1987-2012. Soc Psychiatry Psychiatr Epidemiol. (2019) 54:99–110. 10.1007/s00127-018-1593-z 30171272

[40] Phillips MR Li X Zhang Y. Suicide rates in China, 1995–99. Lancet. (2002) 359:835–40. 10.1016/S0140-6736(02)07954-0 11897283

[41] Gunnell D Appleby L Arensman E Hawton K John A Kapur N. Suicide risk and prevention during the COVID-19 pandemic. Lancet Psychiatry. (2020) 7:468–71. 10.1016/S2215-0366(20)30142-5 32330430 PMC7173821

[42] Sher L. The impact of the COVID-19 pandemic on suicide rates. QJM. (2020) 113:707–12. 10.1093/qjmed/hcaa202 32539153 PMC7313777

[43] Yan Y Hou J Li Q Yu NX. Suicide before and during the COVID-19 pandemic: a systematic review with meta-analysis. Int J Environ Res Public Health. (2023) 20:3346. 10.3390/ijerph20043346 36834037 PMC9960664

[44] Chew KS McCleary R. The spring peak in suicides: a cross-national analysis. Soc Sci Med. (1995) 40:223–30. 10.1016/0277-9536(94)E0070-9 7899934

[45] Woo JM Okusaga O Postolache TT. Seasonality of suicidal behavior. Int J Environ Res Public Health. (2012) 9:531–47. 10.3390/ijerph9020531 22470308 PMC3315262

[46] Yang CT Yip PSF Cha ES Zhang Y. Seasonal changes in suicide in South Korea, 1991 to 2015. PLoS ONE. (2019) 14:e0219048. 10.1371/journal.pone.0219048 31251776 PMC6599115

[47] Atmaca M Kuloglu M Tezcan E Ustundag B Gecici O Firidin B. Serum leptin and cholesterol values in suicide attempters. Neuropsychobiology. (2002) 45:124–7. 10.1159/000054950 11979060

[48] Morken G Lilleeng S Linaker OM. Seasonal variation in suicides and in admissions to hospital for mania and depression. J Affect Disord. (2002) 69:39–45. 10.1016/S0165-0327(00)00373-6 12103450

[49] Vyssoki B Praschak-Rieder N Sonneck G Blüml V Willeit M Kasper S. Effects of sunshine on suicide rates. Compr Psychiatry. (2012) 53:535–9. 10.1016/j.comppsych.2011.06.003 21821241

[50] Vyssoki B Kapusta ND Praschak-Rieder N Dorffner G Willeit M. Direct effect of sunshine on suicide. JAMA Psychiatry. (2014) 71:1231–7. 10.1001/jamapsychiatry.2014.1198 25208208

[51] Fernández-Niño JA Flórez-García VA Astudillo-García CI Rodríguez-Villamizar LA. Weather and suicide: a decade analysis in the five largest capital cities of Colombia. Int J Environ Res Public Health. (2018) 15:1313. 10.3390/ijerph15071313 29932440 PMC6069433

[52] Bando DH Scrivani H Morettin PA Teng CT. Seasonality of suicide in the city of Sao Paulo, Brazil, 1979–2003. Braz J Psychiatry. (2009) 31:101–5. 10.1590/S1516-44462009000200004 19578680

[53] Kok LP Tsoi WF. Season, climate and suicide in Singapore. Med Sci Law. (1993) 33:247–52. 10.1177/002580249303300311 8366788

[54] Cantor CH Hickey PA De Leo D. Seasonal variation in suicide in a predominantly Caucasian tropical/subtropical region of Australia. Psychopathology. (2000) 33:303–6. 10.1159/000029162 11060513

[55] Djembi LF Vaiva G Debien C Duhem S Demarty A-L Koudou Y-A. Changes in the number of suicide re-attempts in a French region since the inception of VigilanS, a regionwide program combining brief contact interventions (BCI) *BMC Psychiatry*. (2020) 20:26. 10.1186/s12888-020-2443-6 31992251 PMC6986096

[56] Hawton K Casañas I Comabella C Haw C Saunders K. Risk factors for suicide in individuals with depression: a systematic review. J Affect Disord. (2013) 147:17–28. 10.1016/j.jad.2013.01.004 23411024

[57] Akkaoui MA Chan-Chee C Laaidi K Fifre G Lejoyeux M Vaiva G. Seasonal changes and decrease of suicides and suicide attempts in France over the last 10 years. Sci Rep. (2022) 12:8231. 10.1038/s41598-022-12215-3 35581322 PMC9114420

[58] McLoughlin AB Gould MS Malone KM. Global trends in teenage suicide: 2003–2014. QJM. (2015) 108:765–80. 10.1093/qjmed/hcv026 25638789

[59] Carballo JJ Llorente C Kehrmann L Flamarique I Zuddas A Purper-Ouakil D. Psychosocial risk factors for suicidality in children and adolescents. Eur Child Adolesc Psychiatry. (2020) 29:759–776. 10.1007/s00787-018-01270-9 30684089 PMC7305074

[60] Zhao S Zhang J. Suicide risks among adolescents and young adults in rural China. Int J Environ Res Public Health. (2014) 12:131–45. 10.3390/ijerph120100131 25546276 PMC4306853

[61] Wu R Zhu H Wang ZJ Jiang CL. A Large Sample Survey of Suicide Risk among University Students in China. BMC Psychiatry. (2021) 21:474. 10.1186/s12888-021-03480-z 34583673 PMC8477567

[62] Li H Fu R Zou Y Cui Y. Predictive roles of three-dimensional psychological pain, psychache, and depression in suicidal ideation among Chinese College Students. Front Psychol. (2017) 8:1550. 10.3389/fpsyg.2017.01550 28955271 PMC5601061

[63] Zeng B Zhao J Zou L Yang X Zhang X Wang W. Depressive symptoms, post-traumatic stress symptoms and suicide risk among graduate students: the mediating influence of emotional regulatory self-efficacy. Psychiatry Res. (2018) 264:224–30. 10.1016/j.psychres.2018.03.022 29655115

[64] Agerbo E Nordentoft M Mortensen PB. Familial, psychiatric, and socioeconomic risk factors for suicide in young people: nested case-control study. BMJ. (2002) 325:74. 10.1136/bmj.325.7355.74 12114236 PMC117126

[65] Zhong BL Chiu HF Conwell Y. Rates and characteristics of elderly suicide in China, 2013-14. J Affect Disord. (2016) 206:273–9. 10.1016/j.jad.2016.09.003 27639861

[66] Wand APF Zhong BL Chiu HFK Draper B De Leo D. COVID-19: the implications for suicide in older adults. Int Psychogeriatr. (2020) 32:1225–30. 10.1017/S1041610220000770 32349837 PMC7235297

[67] Santini ZI Jose PE Cornwell EY Koyanagi A Nielsen L Hinrichsen C. Social disconnectedness, perceived isolation, and symptoms of depression and anxiety among older Americans (NSHAP): a longitudinal mediation analysis. Lancet Public Health. (2020) 5:e62–70. 10.1016/S2468-2667(19)30230-0 31910981

[68] Koo YW Kõlves K De Leo D. Suicide in older adults: differences between the young-old, middle-old, and oldest old. Int Psychogeriatr. (2017) 29:1297–306. 10.1017/S1041610217000618 28511737

[69] Ladwig KH Klupsch D Ruf E Meisinger C Baumert J Erazo N. Sex- and age-related increase in prevalence rates of death wishes and suicidal ideation in the community: results from the KORA-F3 Augsburg Study with 3,154 men and women, 35 to 84 years of age. Psychiatry Res. (2008) 161:248–52. 10.1016/j.psychres.2008.03.011 18817981

[70] Lange S Bagge C Probst C Rehm J. Proportion of individuals with past-year suicidal ideation who attempted suicide over the past 10 years in the United States, and the influence of age and sex. Crisis. (2021) 42:152–6. 10.1027/0227-5910/a000690 32431200

[71] Choi M Lim J Chang S-S Hwang M Kim C-S Ki M. Financial hardship and suicide ideation: age and gender difference in a Korean panel study. J Affect Disord. (2021) 294:889–96. 10.1016/j.jad.2021.07.102 34375217

[72] Choi SB Lee W Yoon JH Won JU Kim DW. Risk factors of suicide attempt among people with suicidal ideation in South Korea: a cross-sectional study. BMC Public Health. (2017) 17:579. 10.1186/s12889-017-4491-5 28619107 PMC5472995

[73] Bommersbach TJ Rosenheck RA Petrakis IL Rhee TG. Why are women more likely to attempt suicide than men? Analysis of lifetime suicide attempts among US adults in a nationally representative sample. J Affect Disord. (2022) 311:157–64. 10.1016/j.jad.2022.05.096 35598742

[74] Richardson C Robb KA McManus S O'Connor RC. Psychosocial factors that distinguish between men and women who have suicidal thoughts and attempt suicide: findings from a national probability sample of adults. Psychol Med. (2023) 53:3133–41. 10.1017/S0033291721005195 35012702 PMC10235670

[75] Carretta RF McKee SA Rhee TG. Gender differences in risks of suicide and suicidal behaviors in the USA: a narrative review. Curr Psychiatry Rep. (2023) 25:809–24. 10.1007/s11920-023-01473-1 37930559 PMC11225381

[76] Yang G-H Phillips MR Zhou M-G Wang L-J Zhang Y-P Xu D. Understanding the unique characteristics of suicide in China: national psychological autopsy study. Biomed Environ Sci. (2005) 18:379–89. 10.1159/000082719 16544520

[77] Caine ED. Changing the focus of suicide research in China from rural to urban communities. Shanghai Arch Psychiatry. (2013) 25:174–5. 10.3969/j.issn.1002-0829.2013.03.007 24991153 PMC4054551

[78] Hsieh CR Qin X. Depression hurts, depression costs: the medical spending attributable to depression and depressive symptoms in China. Health Econ. (2018) 27:525–44. 10.1002/hec.3604 28990318

[79] Yu H Nie C Zhou Y Wang X Wang H Shi X. Suicides in China from 2008-2017: a longitudinal epidemological study. Int J Crit Illn Inj Sci. (2020) 10:88–91. 10.4103/IJCIIS.IJCIIS_108_19 32904529 PMC7456286

